# Purchase trends of processed foods and beverages in urban India

**DOI:** 10.1016/j.gfs.2019.05.007

**Published:** 2019-12

**Authors:** Cherry Law, Rosemary Green, Suneetha Kadiyala, Bhavani Shankar, Cécile Knai, Kerry A. Brown, Alan D. Dangour, Laura Cornelsen

**Affiliations:** a Faculty of Public Health & Policy, London School of Hygiene & Tropical Medicine, 15-17 Tavistock Place, London, WC1H 9SH, UK; b Department of Population Health, London School of Hygiene & Tropical Medicine, Keppel Street, London WC1E 7HT, UK; c Centre for Development, Environment and Policy, SOAS University of London, London, WC1H 0XG, UK; d (Honorary) College of Medicine & Health, University of Exeter, Exeter, EX1 2LU, UK

## Abstract

• Unique dataset allows analysis of urban Indian shopping habits of processed foods.
• Beyond staples, purchases of processed foods and beverages for home use are low.
• Fastest rate of growth seen in sweet and salty snacks, and edible oil purchases.
• Highest volume of processed foods and beverages purchased by Delhi population.
• Large variations across states in level and trends of purchases over time.

## 1 Introduction

India, the country with the second largest population in the world, is facing an evolving challenge of a double burden of malnutrition. India ranks 103rd out of 119 low- and middle-income countries in the Global Hunger Index (von Grebmer et al., 2018). On top of the persisting high burden of undernutrition, there is an increasing prevalence of obesity, which is a known risk factor for a range of diseases including several cancers and cardiovascular diseases (CVDs) (World Health Organization, 2018). Between 2005/06 and 2015/16, the prevalence of overweight and obesity grew by nearly 10 percentage points to reach 27.6% among men and 37.6% among women in urban India and 14.8% and 18.2%, respectively, in rural India (Luhar et al., 2018).

This nutritional challenge is further complicated by large heterogeneity across states. From 1998 to 2014, the number of states where prevalence of overweight exceeded 20% increased threefold, from two to six. In some southern states (e.g., Tamil Nadu and Kerala) and in wealthier states (e.g., Punjab and Himachal Pradesh), the percentage of overweight women was greater than those who were underweight (Meenakshi, 2016). Using Global Burden of Disease Study (GBD) data, Prabhakaran et al. (2018) reported an increase in the prevalence of CVDs in every part of India from 1991 to 2016, but demonstrated substantial variations in the burden across states.

One of the leading causes of the burden of disease from CVDs, according to the GBD data, were dietary risks. In 2016 these risks contributed to 51.8% and 59.4% of the total disability-adjusted life years from CVDs among Indian women and men respectively (Prabhakaran et al., 2018). In recent decades, researchers have observed an increased intake of sugar, oils and highly processed food in Indian diets (e.g. Meenakshi, 2016; Misra et al., 2011; Popkin et al., 2001; Shetty, 2013), with more apparent changes identified in urban India (Shetty, 2002; Gulati and Misra, 2014). These changes marked some key dietary shifts in the nutrition transition, which have also been observed in other low- and middle-income countries (Drewnowski and Popkin, 1997; Popkin et al., 2012; Popkin, 2014).

With the rapid growth of the modern global food retail sector, the consumption of packaged and processed foods has become more common in much of the world (Popkin, 2017). In India, the overall per capita sales of packaged and processed foods nearly doubled from USD 31.3 in 2012 to USD 57.7 in 2018 at constant 2018 prices, according to data from Euromonitor (2019). The 2018 Global Nutrition Report emphasised the negative dietary impacts of industrially processed and manufactured foods as they often increase the overall dietary content of sugars, saturated and trans-fat, salt and dietary energy density while decreasing the content of protein, dietary fibre, potassium, iron, zinc, magnesium and other micronutrients (Development Initiatives, 2018). It is thus unsurprising that many studies have raised concerns over the health implications of the rising consumption of these foods in India (as well as in other developing countries) (Baker and Friel, 2014; Moodie et al., 2013; Popkin et al., 2012; Thow et al., 2016). The reasons behind such trends are complex. Literature attributes the nutrition transition often to increased globalization, trade and economic growth as well as to accompanying changes in labour markets and life styles more generally (Thow and Hawkes, 2009; Kearney, 2010; Baker and Friel, 2014).

The aim of this paper is to analyse purchase trends of eight distinct categories of processed foods and beverages between 2013 and 2017. We use a unique, large and demographically representative dataset from 'Kantar - Worldpanel Division, India' on take-home purchases of packaged food and beverages by urban Indian households. The focus on urban areas is motivated by their considerably higher levels of obesity and overweight in comparison to rural areas. In light of the disparities in nutritional outcomes across states as well as the diversity of Indian diet, we also performed a state-level analysis of the purchase trends.

To the best of our knowledge, only a few cross-sectional studies to date have investigated the consumption patterns of processed foods and beverages in India in detail. A systematic review of studies looking at dietary patterns in India identified a diet pattern high in sweets and snacks that was associated with higher diabetes risk (Green et al., 2016). Daniel et al. (2011) found evidence for dietary patterns characterised by higher intakes of fried snacks and sweets in Mumbai and Trivandrum. Satija et al. (2015) identified three distinct dietary patterns among factory workers from Lucknow, Nagpur, Hyderabad and Bangalore, in which two of them were associated with high intake of snacks.

Other existing work on Indian dietary patterns utilised household level data from the National Sample Survey (NSS) and the National Family Health Survey (NFHS). While these cross-section datasets provide detailed records on the consumption of unprocessed foods (grains, meat, fruits etc.), they often do not consistently collect information on quantities of processed foods or beverages consumed. Hence, detailed analyses on the recent trends in consumption patterns of processed foods and beverages in India remain a significant gap in the literature.

Our analysis showed substantial variations in the purchase level of processed foods and beverages across states as well as variability of trends within states over time. We found that beyond dietary staples (processed wheat, oils, milk) per capita take-home purchases of processed food and beverages are relatively low and infrequent, particularly in comparison to middle-income and high-income countries. Sweet snacks, salty snacks, edible oils and ‘other processed foods’ (defined below, but which predominantly consist of noodles) were the main categories of foods where volume of purchases have increased over time whereas soft drink purchases and dairy products beyond milk showed a slight decline. If the rising trends in snack and oil purchases continue, with no further changes in the consumption of other foods, the dietary risks among urban Indian households will change with the potential for higher prevalence of overweight and further challenges to the nutritional health of the Indian population.

## 2 Methods

### 2.1 Data description

The data were provided by 'Kantar - Worldpanel Division, India', a commercial consumer insight company that operates an ongoing, demographically representative panel that provides information on purchases of consumer goods, including packaged foods and beverages. Our dataset covers product-level purchases of all packaged foods and beverages for consumption at home by the urban households on the panel between January 2013 and December 2017. Similar commercial food purchase and sales data have been increasingly used in academic research to measure dietary patterns, estimate nutritional intake, model disease outcomes and evaluate policies, and they are generally considered a good indicator of diets (Bandy et al., 2019). Recent studies, for example, that have used Kantar Worldpanel data on food purchases include, Caillavet et al. (2018) who analysed trends of food purchases of French households and Cornelsen et al. (2019) who used the UK household panel to analyse demand for take-home food purchases.

The panel in India, which has been operating since 1981, had a major update after the 2011 Census to ensure representativeness to the Indian urban population with respect to occupational socio-economic status (see table A1 for description), age of the person responsible for food purchase as well as the state of domicile. Households are invited door-to-door to participate in the panel based on these household characteristics. Representativeness is checked by 'Kantar - Worldpanel Division, India' on an ongoing basis and new households are invited where drop out occurs. In our data around 95% of the surveyed households were present in the panel in each year. Despite drop out of 5% of participants, sample distribution of socio-economic classes remained stable across years (Table A2 in the appendix).

The primary shoppers of the participating households fill in paper diaries to record all take-home purchases by volume, which exclude gifts, free samples or home-made products. To ensure that purchases are recorded correctly, interviewers from 'Kantar - Worldpanel Division, India' regularly check the information in the paper diaries against packaging and wrappers that are collected by households in pre-provided containers. The data recorded covers only the volume of purchases and does not include information on monetary expenditure. The dataset includes information on purchases that are taken home and excludes purchases for out-of-home consumption.

The panel covers urban households from 16 major states and Guwahati, a major city in Assam (Table 1). Given the lack of data on state level average incomes over time, we classified the 16 states into three groups based on the share of urban population living below poverty line in 2011–12 as a proxy for income level. This poverty line is estimated by the Indian Government based on private household consumer expenditure over a basket of essential goods (Government of India, 2014). States were considered as low-income if over 20% of their urban population were under the poverty line, middle-income if the percentage was between 10% and 20% and high-income if it was less than 10%.

**Table 1:** Sample distribution and the population below poverty line across states in urban India.

	Number households in the panel in 2013		% of urban population below poverty line in 2011–12
**High urban income states**			
Kerala	2975	(5%)	5.0
Andhra Pradesh	4492	(7%)	5.8
Tamil Nadu	5699	(9%)	6.5
Maharashtra	7822	(12%)	9.1
Delhi	2516	(4%)	9.8
**Middle urban income states**			
Gujarat	3855	(6%)	10.1
Punjab/Haryanaˆ	4516	(7%)	9.2/10.3
Rajasthan	2551	(4%)	10.7
West Bengal	4203	(6%)	14.7
Karnataka	4390	(7%)	15.3
Orissa	1825	(3%)	17.3
**Low urban income states**			
Madhya Pradesh	5521	(9%)	21.0
Jharkhand	3382	(5%)	24.8
Chhattisgarh	1309	(2%)	24.8
Uttar Pradesh	6477	(10%)	26.1
Bihar	2362	(4%)	31.2
Guwahati*	1046	(2%)	n/a

Note: Figures in parentheses give the percentage of total sample in 2013. ˆThese two states are not separated in the data. *Guwahati is excluded in the state-level analysis as it is only part of the urban sector of Assam.

In total, our dataset consists of 78,320 unique urban households across five years and the sample size of each year ranges from 64,941 to 69,035 households of the panel (Table 2).

**Table 2:** Sample size across years.

	2013	2014	2015	2016	2017
Total number of households	64,941	69,035	67,523	67,865	66,574
Households from previous year	n/a	61,873 (90%)	66,260 (98%)	64,657 (95%)	63,900 (96%)
New households	n/a	7162 (10%)	1263 (2%)	3208 (5%)	2674 (4%)

*Corresponding percentages to the full sample are given in parenthesis.

### 2.2 Food groups

Due to significant variety of products recorded in the data, analysing purchase trends at product level would make it difficult to understand the overall changes in the purchases of processed foods and beverages. With consideration of the products’ properties (sweet, savory) and modes of consumption (e.g. major ingredient, snack or part of a meal or cooking process), we separated individual products into the eight food categories outlined below. As we did not have information on the nutrient content of each of the individual products, we could not distinguish between healthier and less healthy products within each group.

**Sweet snacks**: chocolate, honey, biscuits, cookies, jams, peanut butter, chocolate spread, rusk (excludes prepared sweets and cakes)
**Salty snacks**: crackers, potato chips, banana chips, other salty snacks;
**Soft drinks**: carbonated drinks, juices, milk-based drinks, squashes and powdered drinks;
**Milk**: liquid milk and milk powder;
**Dairy products**: butter, *ghee (clarified butter*) and cheese;
**Edible oils**: edible oils and *vanaspati (partially hydrogenated vegetable oil);*
**Processed wheat**: *atta (wheat flour),* bread, vermicelli and pasta;
**Other processed foods**: soup, ready-to-eat meal, ready-to-cook mix, frozen food, breakfast cereals, noodles, ketchup, table sauces.

Each of the eight food groups has at least 1 million purchase records. More information on the number of observations and examples of food items in each food group is given in table A3 in the appendix.

### 2.3 Estimation of per capita purchase volume

To estimate the population-level volume of per capita purchases of each food group in each period (quarter or year), we first estimate weighted total urban market purchases during the period in a food group and then apply population size estimates to derive per capita purchase. We obtained the urban population figures using the latest official census data released in 2011, in each quarter from 2013 to 2017 in two steps. First, we computed the quarterly average population growth rates ($r_{s})$ using Indian Census in 2001 and 2011 with the formula: $r_{s}={\left(\frac{pop_{s,\;2011}}{pop_{s,\;2001}}\right)}^{1/40}-1$ , where $pop_{s}$ is the population size of state s in the corresponding census (i.e. 2001 or 2011) and the denominator in the power term indicates the number of quarters between 2001 and 2011. We then projected these growth rates forward from 2011 to estimate the population size for each state in each quarter up to 2017 with the assumption that the average growth rates remained the same across quarters. The total population of urban India was obtained by summing up the population figures of each state in each quarter.

Quarterly trends were estimated by a linear ordinary least squares regression of purchase quantities on time dummies. For easier interpretation, annual purchases of processed foods rather than quarterly purchases are presented in the state-level analysis.

## 3 Purchase trends of processed foods and beverages in urban India

**Fig. 1:**
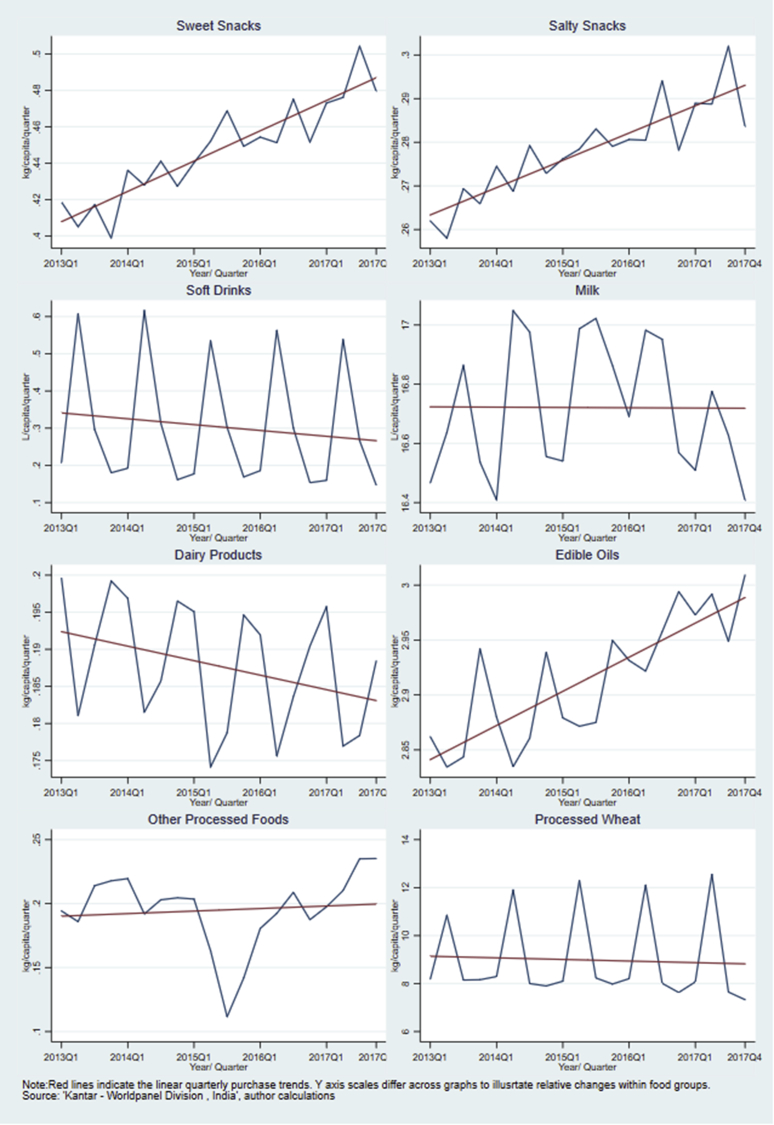
Quarterly purchase patterns of processed foods and beverages in urban India, 2013-17.

Fig. 1 shows the quarterly per capita purchase volume across the food groups. Further information is provided in the appendix, where table A4 summarises the estimated level of annual per capita purchases in 2013 and 2017 and table A5 gives both the quarterly average and the annual percentage of households who reported purchase in each year.

In Fig. 1, sweet snacks and salty snacks display a clear increasing trend in take-home purchases with marginal variation seasonally. On average, the per capita annual purchase was 1.64 kg of sweet snacks and 1.06 kg of salty snacks in 2013. By 2017, this rose to 1.93 kg (17%) for sweet snacks and 1.16 kg (9%) for salty snacks. Purchases from both food groups were also relatively common, with 80–89% of the respondents reporting purchases of products from either of the groups at least once per quarter over the years.

Edible oils also showed an increasing trend in per capita purchases between 2013 and 2017 (by 0.44 kg, 4%), consistent with the rise in share of calorie intakes from oils and fats identified in the NSS report on nutritional intake (NSSO, 2014). Oil purchases showed relatively large quarterly fluctuations, particularly in 2014 and 2015, with almost a 0.1 kg difference in per capita purchases between the first and fourth quarters of the year. This fluctuation appears to flatten over time.

The level of take-home purchases of soft drinks was low at only 1.29 L per capita per year in 2013 which decreased to 1.11 L (14%) by 2017. The purchases were also less common than other food groups, with on average 46% of households reporting a quarterly purchase of soft drinks and around 70% reporting a purchase at least once a year. Demand for soft drinks fluctuated seasonally by around 400 ml, peaking during the hottest months of the year (i.e. second quarter). The highest level of quarterly take-home purchases was recorded in the second quarter in 2014 at 0.6 L per capita.

Milk was the most widely purchased group. Over 96% of surveyed households reported at least one purchase of milk quarterly and 99% purchased milk annually. For each quarter, each person purchased on average 16–17 L of milk for home consumption, which was equivalent to over 64 L of per capita milk purchase per year. Despite seasonal fluctuations by 200–400 ml, the overall trend was stable. In contrast, there was a decreasing trend in the purchases of the other dairy products and the total annual purchase was low (less than 0.77 kg per capita), including around 28% of respondents who did not purchase any other dairy products during a year.

Processed wheat displayed a relatively stable purchase trend but growing quarterly fluctuation with more purchases made, on average, during the second quarter of the year. In 2013, the per capita purchase in the second quarter was 10.85 kg, around 2.69 kg higher than in the fourth quarter. This difference rose to 4.32 kg in 2015 and to 5.23 kg by 2017. It should be noted that these figures are likely to underestimate the total purchase of processed wheat products as unpackaged wheat flour is not captured by the data.

**Fig. A1:**
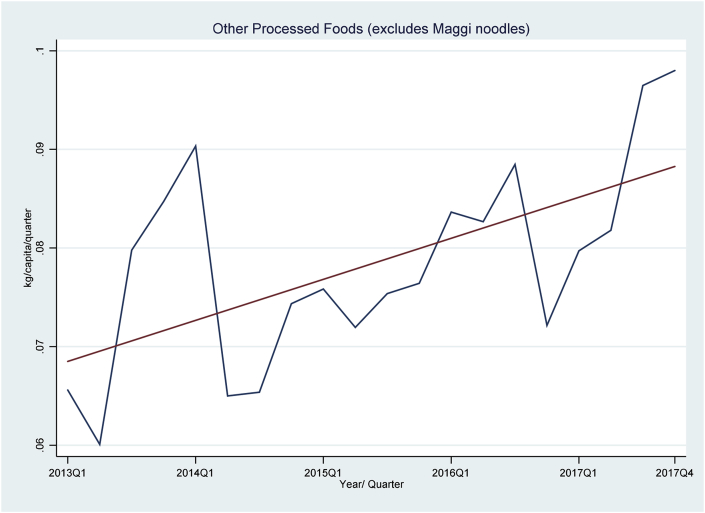
Quarterly purchase patterns of other processed foods (excluding Maggi noodles) in urban India, 2013–17.

On average, 73% of the respondents purchased ‘other processed foods’ at least quarterly. While the overall volume was low but with a slightly increasing trend, there was a sharp drop in 2015. This was driven largely by the temporary nationwide ban on the sale of Maggi noodles over food safety concerns in June 2015 (The Times of India, 2017). Overall, Maggi noodles account for more than half of the volume of ‘other processed foods’ bought (0.52 kg in 2013). Despite the 2015 decline, a 9% increase was identified in ‘other processed food’ purchases between 2013 and 2017. Once Maggi noodles were excluded, the rate of increase rose to 22% during the same period. (Appendix, figure A1).

## 4 State level heterogeneity in purchases of processed foods and beverages

**Fig. 2:**
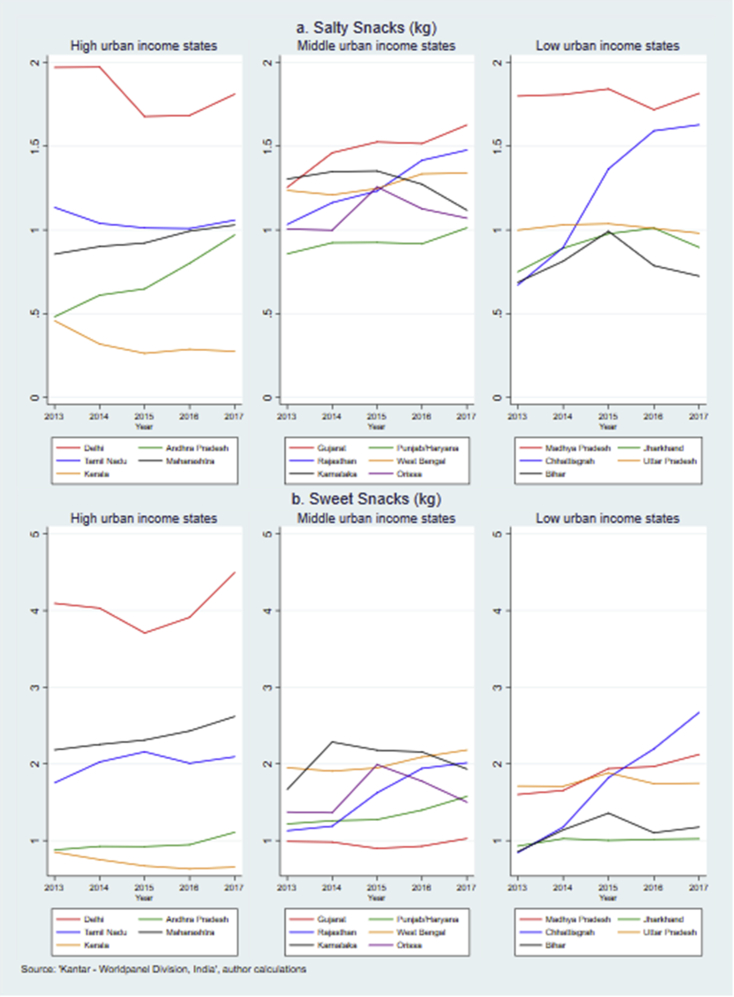
Annual per capita purchase of sweet and salty snacks across Indian states, 2013–2017.

**Fig. 3:**
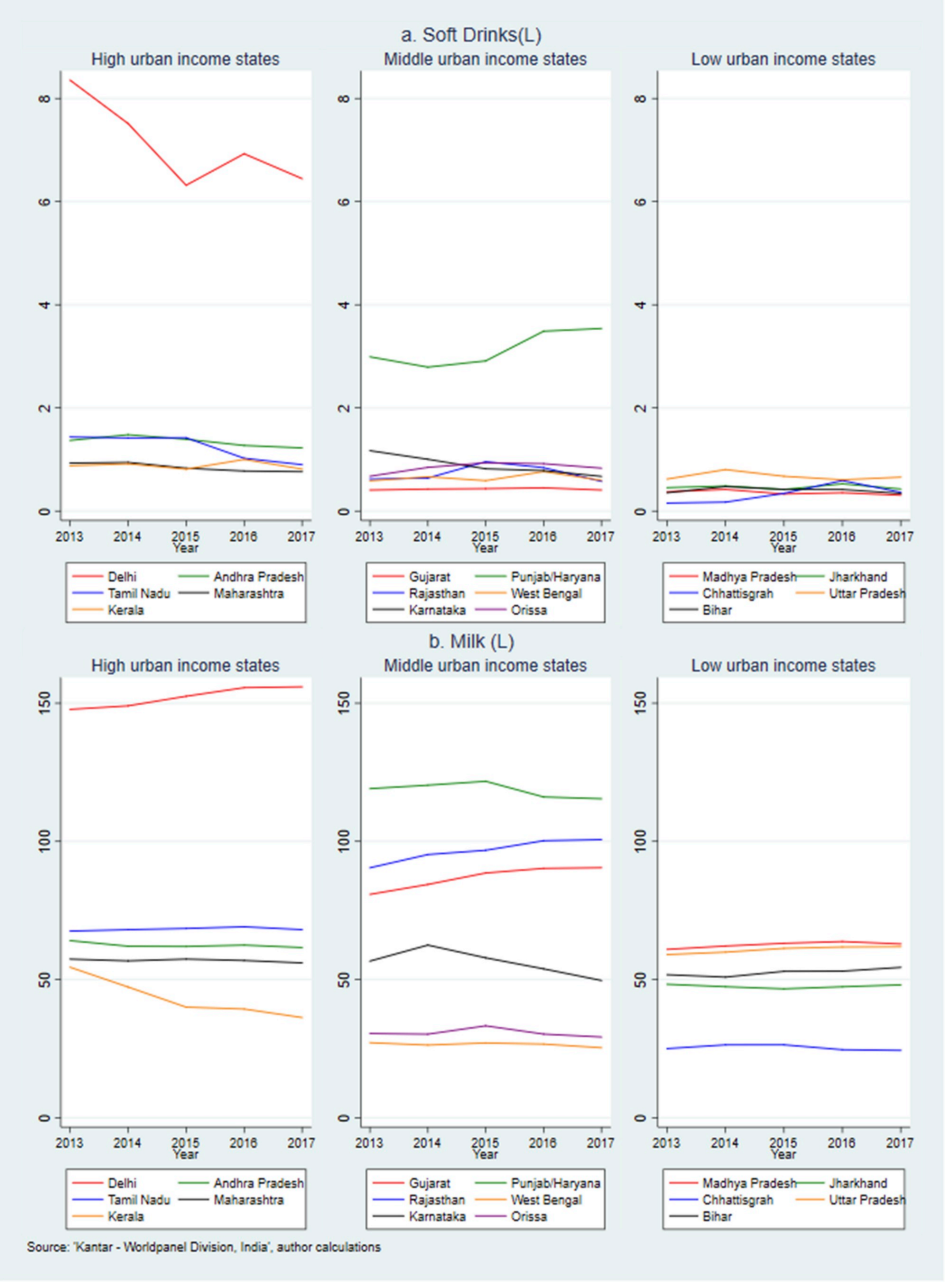
Annual per capita purchase of soft drinks and milk across Indian states, 2013–2017.

**Fig. 4:**
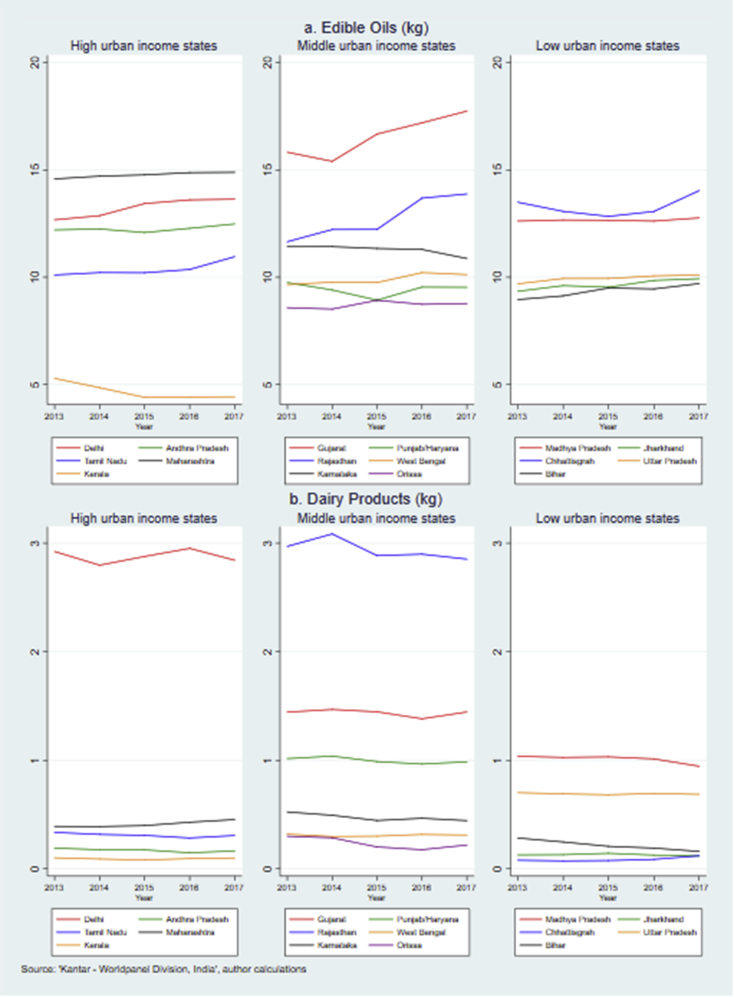
Annual per capita purchase of edible oils and dairy products across Indian states, 2013–2017.

**Fig. 5:**
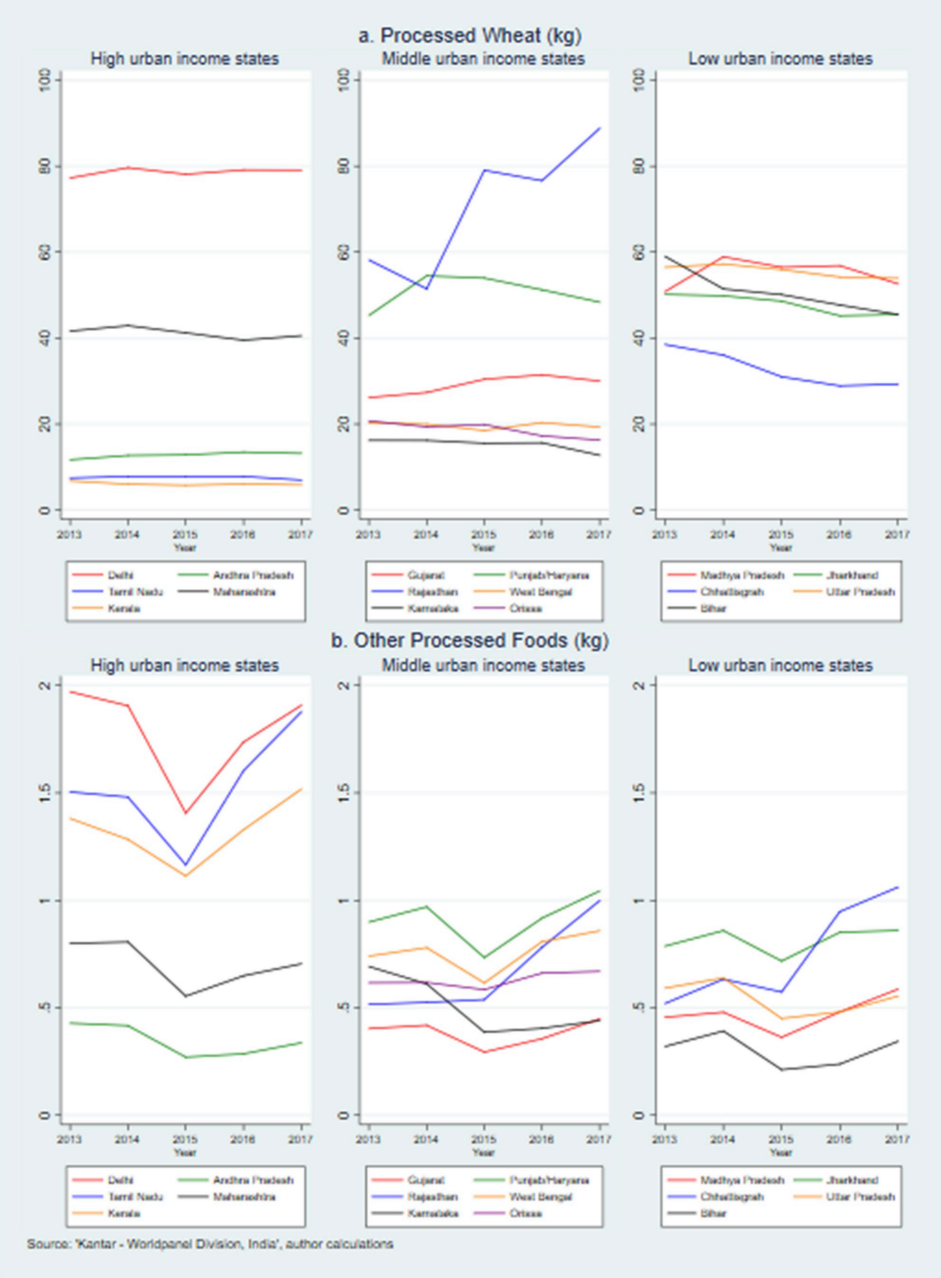
Annual per capita purchase of processed wheat and ‘other processed foods’ across Indian states, 2013–2017.

Substantial state level differences in the take-home purchases of processed foods and beverages were observed (Fig. 2, Fig. 3, Fig. 4, Fig. 5). Consistent with the trends highlighted in section 3, Fig. 2a and b shows that most states experienced a rise in take-home purchases of salty snacks (n = 10 out of 16 states) and sweet snacks (n = 12). Kerala had the lowest level of annual purchases of both snacks and to the contrary of more than half of the states, saw a decreasing trend. From 2013 to 2017, the per capita purchase of salty snacks in Kerala dropped by 41% from 0.46 kg to 0.27 kg while that of sweet snacks declined by 23% from 0.85 kg to 0.65 kg. The level of salty snacks bought for home consumption also decreased in Delhi, Karnataka and Tamil Nadu. The level of sweet snacks purchases was far higher in Delhi than in any of the other states (around 4 kg per year). The purchase level of salty snacks did not differ across states by level of poverty, although the middle-income states showed most consistent rise in purchases. A similar pattern was also seen for sweet snacks with the exception of Delhi where purchase level (4–4.5 kg) was twice the level of remaining states (1–2.5 kg).

Turning to Fig. 3a, the top panel shows that wealthier states in general purchased relatively more soft drinks than those in the middle and low-income states, with the predominant difference being between Delhi and the rest of the states. While most states had a stable purchase pattern of soft drinks, Delhi displayed a 23% drop in per capita purchase (1.91 L) over the five years. During the same period, Punjab/Haryana experienced an 18% (0.57 L) increase in soft drinks brought per capita. In rest of the states the average purchases were below 2 L per year and had less prominent trends.

Fig. 3b demonstrates large variations in milk purchases across Indian states with the highest level in Delhi (annual purchases of 155 L per capita in 2017), compared to the lowest level in Chhattisgarh (24 L in 2017). The largest reduction in packaged milk purchases was in Kerala, declining from 55 L per capita in 2013 to 36 L in 2017. Karnataka also slowed a decreasing trend starting from 2014. At the same time, Delhi, Rajasthan and Gujarat increased their take-home purchases of milk by around 10 L per capita over the five years.

Kerala had the lowest level of per capita purchases of edible oils (Fig. 4a) (<4.5 kg in 2017). Compared to other food groups, there was a smaller gap between the edible oil purchase between Delhi and other states. The purchase level increased fastest in Rajasthan (19%) and Gujarat (12%). In particular, Gujarat also bought the highest volume of edible oils per capita over the period of interest (17.74 kg in 2017). Only a few states purchased more than 1 kg dairy products (other than milk) per capita a year (Fig. 4b). The level was highest in Rajasthan and Delhi (2.97 kg and 2.92 kg in 2013), followed by Gujarat (1.44 kg in 2013). For most states, the purchase pattern of dairy products was stable. In Kerala and Chhattisgarh, less than 0.13 kg dairy products were purchased per capita per year.

Purchase patterns of processed wheat are relatively stable in high-income states (Fig. 5a) but slightly downward in low-income states. Among all states, Rajasthan experienced the fastest increase in purchase of processed wheat over five years with estimated annual per capita purchases reaching 89 kg in 2017, exceeding that of Delhi (79 kg). Lastly, Fig. 5b shows the per capita purchase trends of ‘other processed foods’. In line with Fig. 1, most states display a sharp drop in 2015 although the decline is more apparent in higher-income states; particularly those with relatively higher levels of purchases (Delhi and Tamil Nadu). In absolute terms, Delhi experienced the biggest annual decrease of ‘other processed food’ purchases, from 1.91 kg in 2014 to 1.41 kg in 2015 (26%). In Rajasthan and Chhattisgarh a rapid increase was seen in the last two years as purchase level doubled by 2017.

## 5 Discussion

Processed foods have risen to public health policy focus because of concerns over their impacts on health (e.g. beverages, snacks and ready meals are often high in sugar, fat and/or salt content) and their consumption is identified as a risk factor for obesity and several non-communicable diseases. Our analysis of take-home purchases of packaged and processed foods in urban India showed that, with the exception of staples such as packaged milk, processed wheat or edible oils, purchases of processed foods and beverages on average, are still relatively low at population level.

The per capita level of salty snack purchases remains low in urban India (1.1 kg in 2017) in comparison to the US and the UK where it reached 9.5 kg and 7 kg respectively in 2015 (IFT, 2016). Similarly, the level of sweet biscuit purchases was much higher in Western Europe and the US (5.9 kg and 6.3 kg per capita respectively in 2017) than in urban India (1.9 kg in 2017). Comparing to other middle income countries, the level of savory snack purchases in urban India was higher than in Pakistan (0.2 kg per capita) or Vietnam (0.8 kg) but lower than in Thailand (1.5 kg) or China (1.8 kg) in 2017 (Euromonitor, 2019). For sweet snacks, the level of purchase in urban India was similar to China, Vietnam and Pakistan (1.3–1.5 kg per capita in 2017) but higher than in Thailand (0.7 kg) (Euromonitor, 2019). The overall level of purchases of soft drinks was also low (1.1 L per capita in 2017) in comparison to the average per capita annual sales of 20.9 L in lower middle income countries (Monteiro et al., 2013) and much lower when comparing to high-consumption countries such as Mexico where 231.2 L of soft drinks were sold per capita in 2017 (Euromonitor, 2019).

Sweet and salty snacks as well as edible oils were the main food groups for which volume of purchases increased over time. Purchases of ‘other processed foods’, which predominantly consist of noodles, also showed an increasing trend especially if the sharp decline driven by food safety concerns in Maggi Noodles in 2015 is excluded (figure A1). These trends are in general support of a nutrition transition taking place in India, demonstrating higher processed food consumption along with income growth (5% per year between 2013 and 2017 (World Bank, 2019)) and greater urbanisation (Popkin, 2006; Popkin and Ng, 2007; Hawkes et al., 2017). In addition to growing wealth, access to processed foods is also facilitated by the rising number of modern grocery retailers (Hawkes et al., 2017) which increased by 13.9% between 2013 and 2017 (Euromonitor, 2019).

The rising trend in purchases of snacks, edible oils and ‘other processed foods’ might also have been driven by the growing Indian policy focus on promoting food processing as well as liberalization of foreign direct investment in food processing and retailing which enhanced the availability and affordability of processed foods (Thow et al., 2016). Furthermore, literature on the nutrition transition suggests that trade liberalization, food industry marketing, expansion of the global mass media, increasing female economic participation and changes in activity patterns are other factors that might have contributed to the rising processed food consumption (Kearney, 2010; Baker and Friel, 2014). Considering the complexity of these drivers, as well as heterogeneity in trends observed, it is clear that detailed empirical analyses are needed to disentangle their roles in driving the trends in urban India.

It is also clear that the upward trend in purchases snack foods is likely to contribute to the rising dietary risk of disease. Equally, the growing level of edible oil purchases is a potential health concern as oils can increase energy density of foods consumed which is a risk factors to obesity and NCDs. For example, a diet with higher intakes of butter, hydrogenated oil, ghee, vegetable oil, mustard oil, fish, high-fat dairy products and refined grain was found to be associated with an increased risk of general as well as of central obesity in women in West Bengal (Ganguli et al., 2011).

Kerala, the most literate state in India (as per the Census of India, 2011), was the only state displaying decline in both sweet and salty snacks purchases by urban households. These changes might be explained by a greater awareness of the health impacts of snack consumption due to the higher general education level of the population; as well as the public health focus of the forward thinking Keralan state government: NITI Aayog ranked Kerala as the top performing Indian state in the health sector due to its sustained focus on health and consistent performance on several health outcomes (NITI Aayog, 2018). Recent state level dietary health interventions have particularly focused on reducing dietary related NCD health outcomes. For example, a 14.5% `fat’ tax on junk food was announced in 2016 (The Economic Times, 2016) and most recently the Keralan government is developing an action plan to combat trans-fat consumption, with the support of WHO and the FSSAI (The New Indian Express, 2019).

One key finding of this paper is the slight decrease in take-home purchases of soft drinks in urban India, especially in Delhi and Tamil Nadu which are the wealthier states. Soft drinks have in particular been targeted by policies in an attempt to reduce their consumption and thus sugar intake. Unless purchases for consumption out-of-home, which these data do not capture, have an opposite trend, the slight reduction in soft drink purchases for home consumption observed is encouraging for reducing the risk of obesity in those states (World Health Organization, 2003). In contrast, the evidence of increased soft drink purchases in Punjab/Haryana and Chhattisgarh may exacerbate the nutritional challenges in these regions. In 2017, the Indian government imposed a 28% Goods and Service tax rate with 12% additional tax levied on aerated drinks (Government of India, 2017b; Government of India, 2017a). This tax on sugary drinks, if sustained, could help potentially combat any rising intake of soft drinks. However, to fully understand the potential of the tax, further information is needed on added sugar consumption from other sources (e.g. hot beverages, home-made beverages such as juice or lemonade, as well as from foods).

With the exception of soft drinks, the state-level disparity in packaged food purchases does not seem to be related to the income status of states (measured according to share of population living under the Government defined poverty line), which warrants further analysis of alternative measures for incomes, as well as other structural drivers such as production, trade and foreign investment, in addition to cultural differences. For example, local dietary preferences could help explain why the purchases of processed wheat were higher in Rajasthan than Kerala as wheat is the basic meal in North India while rice is the key staple in the Southern regions (Sen, 2004). Difference in staple foods may also lead to variations in cooking methods across states and hence contribute to the state-level disparity in edible oil purchases (Gulati and Misra, 2017).

### 5.1 Strengths and limitations

Our study has a number of strengths. First, the data come from a large and longitudinal, demographically representative sample of households. Retention of the households in the panel is high with only 5% of households drop-out and are replaced periodically. Product level data also allow flexibility in creating food groups. Second, no other data source covers this range of products across states and over time. Other nationwide household surveys are often limited in the level of information collected in regard to packaged and processed foods and beverages while dietary surveys with more detailed consumption records of these foods tend to be limited in geographical coverage. More importantly, these surveys only provide cross-sectional data on over a short time period and therefore may not be as suitable to study changes in the dietary patterns over time.

There are some limitations to consider when interpreting the findings of this study. First, given that these data have not, to our knowledge, been used in academic research, we compared our estimates of per capita annual purchases in 2013 with those obtained from the latest round of NSS data (2011–12), which is the official government survey on food expenditures on all food items consumed in the last 30 days. We selected food items that were easily identifiable and comparable in both datasets. From table A6 in the appendix, it can be seen that for some food items, i.e. cold beverages, milk (in liquid form) and edible oils, the discrepancy between the estimates from our data and that from NSS data is small (i.e. around 10%), but for other food items, notably butter, the former estimates appear to be considerably higher. This could be due to: i) an increase in consumption of these foods in 2013; ii) an underestimation from NSS data as it only covers 30 days and purchase of dairy products may not take place sufficiently frequently; iii) an overestimation bias in the data from ‘Kantar – Worldpanel Division, India’. The latter is relatively less likely as purchase records were checked against the wrappers of the purchases regularly by interviewers.

Second, the data excludes food purchases made for out-of-home and on-the-go consumption. According to market data from the Euromonitor, the number of food service units in India has grown by 19.5% between 2012 and 2017, including a 27.5% increase in the number of fast food units. Number of transactions grew in food service by 31.7% including in fast food outlets by 48.2% (Euromonitor, 2019). While more detailed research is lacking in this area, with rising income, replacing home-cooked meals with food prepared and eaten out-of-home, is forecasted to be a growing trend in particular among young adults (Euromonitor, 2019).

Third, we had to project urban population size estimates as official data are unavailable beyond 2011. Population trends, particularly in urban areas, can change rapidly and therefore affect our estimation of per capita purchase trends. In this regard, we compared our population projections of urban India from 2013 to 2017 with those projected by the World Bank (see table A7 in the appendix) and found less than 3% difference in estimates which shows accuracy in the methods applied for projections. However, this does make state-level analysis somewhat more susceptible to bias as population growth can vary to a greater extent depending on changes in birth and death rates as well as inter-state migration. Similarly, rapid changes in poverty levels by state over five years could have affected the comparison of purchases across the income-level of states and could explain a lack of clear patterns.

Lastly, while the purchase trends of processed foods and beverages vary greatly across states and are indicative of some changes in the dietary patterns at population level, it is difficult to directly link these to nutrition or health outcomes. A plethora of multi-dimensional factors influence a particular NCD aetiology and pathogenesis and greater information (e.g., dietary intake, nutrient status and health status) would be required at an individual or local community level to complete these analyses.

### 5.2 Further research and conclusion

Further research is needed to identify the drivers of state-specific trends because one-size-fits-all national picture is unlikely to be sufficiently informative for nutrition-related programming and policymaking in India (Cavatorta et al., 2015). Another fruitful avenue would be to analyse how the trends and levels of processed food purchases differ across socio-economic classes and other demographic characteristics since wealthier people tend to allocate their food expenditure differently from poorer people (Deaton and Drèze, 2009; Borkotoky and Unisa, 2018). Finally, it is also important to investigate the purchase trends among sub-populations of regular consumers of packaged and processed foods, their socio-demographic characteristics and possible linkages to dietary and health outcomes.

In conclusion, our analysis showed varying levels of packaged and processed food purchases as well as heterogeneous patterns over time and across the urban areas of the Indian states. Beyond dietary staples (wheat, oil, milk) the level of purchases of packaged and processed foods and beverages was low. However, the rapidly rising level of purchases of sweet snacks, salty snacks, edible oils and ‘other processed foods’, contributing to increasing intake of sugar, salt and fats, are of concern from public health perspective and may require further policy efforts to combat against (Moodie et al., 2013; Monteiro et al., 2013). With no further changes in the consumption of other foods, the dietary risks among urban Indian households might increase, leading to higher prevalence of overweight and thus further complicating the nutritional challenges faced by the country. Nonetheless, policies targeting processed foods alone are not sufficient to tackle all food security challenges in India (Pingali et al., 2017). Improving the nutrient quality of the food system and ensuring equity of access and availability to nutrient rich unprocessed foods remains a key to ending malnutrition in all its forms.

## Funding

This study forms part of the Sustainable and Healthy Food Systems (SHEFS) programme supported by the Wellcome Trust’s Our Planet, Our Health programme [grant number: 205200/Z/16/Z]. LC is funded via UK Medical Research Council Fellowship MR/P021999/1. Funding bodies had no role in the data collection, analysis or interpretation, and no role in the study design or in writing the manuscript.

## Figures and Tables

**Table A1:** Sample distribution of socio-economic class, 2013–2017

Classes	Definitions
Upper class	Minimum literacy/school up to 4 years & minimum 6 durables in their household
Upper middle class	Minimum literacy/school up to 4 years & minimum 5 durables in their household
Middle class	Minimum literacy/school up to 4 years & minimum 3 durables in their household
Lower class	Illiterate and no durable or minimum 1 durable in their home

**Table A2:** Definitions of socio-economic classes

	2013		2014		2015		2016		2017	
Upper class	24527	(38%)	25295	(37%)	24619	(36%)	25578	(38%)	25591	(38%)
Upper middle class	18709	(29%)	19127	(28%)	18745	(28%)	18538	(27%)	17977	(27%)
Middle class	14884	(23%)	15260	(22%)	15017	(22%)	14723	(22%)	14353	(22%)
Lower class	6821	(11%)	9354	(14%)	9144	(14%)	9026	(13%)	8655	(13%)

Note: Figures in parentheses give the percentage of total sample. Households are classified into different SEC groups based on the education level of the primary shopper and the number of assets possessed by the household (Refer to table A2).

**Table A3:** Number of observations and food item examples of each food group

Food Groups	No of purchase records	Food items	Product examples
Sweet snacks	4,686,606	Chocolate	Cadbury Dairy Milk Chocolate
		Honey	Apis Himalaya Honey
		Biscuits	Treff Marie Special
		Jams	Kissan Fruity Mango
		Peanut butter	American Garden Creamy
		Chocolate spread	Pillsbury Chocolate Spread
		Rusk	Baker Street Jeera Rusk
Salty snacks	14,423,632	Crackers	Royal Krack Krackar
		Banana chips	Sri Annai Banana Chips
		Potato chips	Bingo Red Chilli Bijli
		Other salty snacks	Crax Natkhat Masala
Soft drinks	2,341,924	Carbonated drinks	Pepsi Cola Pet Bottle
		Juices	Tropicana 100% Orange
		Milk based drinks	Nestle Nesquik Chocolate
		Squashes and powdered drinks	Rasna Fruit Plus Orange
Milk	107,048,570	Liquid milk	Cavin's Toned Milk
		Milk powder	Sunfresh Dairy Whitener
Edible oils	7,582,521	Edible oils	Aadhar Sunflower Oil
		vanaspati	Dalda Vanaspati
Dairy products	1,868,296	Butter	Amul Yellow Butter
		Ghee (Clarified butter)	Hatsun Ghee (Pouch)
		Cheese	Britannia Milkman Mozzarella
Processed wheat	11,696,969	Atta	Aashirvaad Multigrain Atta
		Bread	Bonn Premium Bread
		Vermicelli	Tasty Treat Vermicelli
		Pasta	Bambino Cheese Pasta
Other processed foods	5,195,869	Soup	Knorr Mushroom Soup
		Ready to eat meal	Kohinoor Paneer Makhani
		Ready to cook mix	Aachi Biryani Rice Mix
		Frozen food	Safal Green Peas
		Breakfast cereals	Horlicks Oats
		Noodles	Maggi Masala Noodles
		Ketchup	Reliance Tomato Ketchup
		Table sauces∗	Ching's Chilli Sauce

∗ Table sauces do not include Indian pickles such as chutneys.

**Table A4:** Estimated per capita annual purchase of food groups, by states, 2013–2017

		Sweet Snacks	Salty Snacks	Soft Drinks	Milk	Dairy Product	Edible Oils	Processed Wheat	Other Processed Foods
		(KG)	(KG)	(L)	(L)	(KG)	(KG)	(KG)	(KG)
All-urban	2013	1.64	1.06	1.29	66.51	0.77	11.48	35.43	0.81
	2017	1.93	1.16	1.11	66.32	0.74	11.92	35.71	0.88
		18%	9%	−14%	0%	−4%	4%	1%	9%
Kerala	2013	0.85	0.46	0.88	54.46	0.10	5.29	7.10	1.38
	2017	0.65	0.27	0.82	36.26	0.10	4.43	6.19	1.52
		−24%	−41%	−7%	−33%	0%	−16%	−13%	10%
Andhra Pradesh	2013	0.88	0.48	1.37	64.10	0.19	12.20	11.74	0.43
	2017	1.11	0.97	1.23	61.59	0.17	12.48	13.23	0.34
		26%	102%	−10%	−4%	−11%	2%	13%	−21%
Tamil Nadu	2013	1.75	1.13	1.44	67.53	0.34	10.11	7.55	1.50
	2017	2.09	1.06	0.90	68.07	0.31	10.95	7.04	1.88
		19%	−6%	−38%	1%	−9%	8%	−7%	25%
Maharashtra	2013	2.18	0.86	0.93	57.40	0.39	14.58	41.68	0.80
	2017	2.62	1.03	0.76	56.04	0.45	14.89	40.60	0.70
		20%	20%	−18%	−2%	15%	2%	−3%	−13%
Delhi	2013	4.10	1.97	8.36	147.78	2.92	12.67	77.57	1.97
	2017	4.50	1.81	6.45	155.93	2.84	13.63	79.46	1.91
		10%	−8%	−23%	6%	−3%	8%	2%	−3%
Gujarat	2013	0.99	1.25	0.41	80.83	1.44	15.82	26.18	0.40
	2017	1.03	1.63	0.41	90.48	1.44	17.74	30.02	0.45
		4%	30%	0%	12%	0%	12%	15%	13%
Punjab/Haryana	2013	1.22	0.86	2.99	119.09	1.01	9.75	45.42	0.90
	2017	1.58	1.01	3.54	115.43	0.99	9.53	48.61	1.04
		30%	17%	18%	−3%	−2%	−2%	7%	16%
Rajasthan	2013	1.13	1.03	0.62	90.45	2.97	11.65	58.26	0.52
	2017	2.01	1.48	0.58	100.65	2.85	13.88	88.94	1.00
		78%	44%	−6%	11%	−4%	19%	53%	92%
West Bengal	2013	1.95	1.24	0.59	27.15	0.32	9.66	20.36	0.74
	2017	2.18	1.34	0.60	25.34	0.31	10.12	19.40	0.86
		12%	8%	2%	−7%	−3%	5%	−5%	16%
Karnataka	2013	1.67	1.30	1.17	56.69	0.52	11.42	16.27	0.69
	2017	1.93	1.12	0.67	49.67	0.44	10.87	12.78	0.44
		16%	−14%	−43%	−12%	−15%	−5%	−21%	−36%
Orissa	2013	1.37	1.01	0.68	30.48	0.30	8.58	20.74	0.62
	2017	1.50	1.07	0.83	29.22	0.22	8.77	16.31	0.67
		9%	6%	22%	−4%	−27%	2%	−21%	8%
Madhya Pradesh	2013	1.60	1.80	0.38	60.92	1.04	12.61	50.91	0.46
	2017	2.12	1.82	0.31	62.90	0.94	12.76	52.75	0.59
		33%	1%	−18%	3%	−10%	1%	4%	28%
Jharkhand	2013	0.93	0.75	0.45	48.29	0.13	9.34	50.21	0.79
	2017	1.02	0.90	0.43	48.08	0.12	9.93	45.55	0.86
		10%	20%	−4%	0%	−8%	6%	−9%	9%
Chhattisgarh	2013	0.84	0.67	0.16	25.03	0.08	13.49	38.56	0.52
	2017	2.67	1.63	0.36	24.37	0.12	14.03	29.41	1.06
		218%	143%	125%	−3%	50%	4%	−24%	104%
Uttar Pradesh	2013	1.71	1.00	0.62	59.04	0.70	9.69	56.60	0.59
	2017	1.75	0.98	0.66	61.91	0.69	10.10	54.07	0.55
		2%	−2%	6%	5%	−1%	4%	−4%	−7%
Bihar	2013	0.86	0.69	0.36	51.75	0.28	8.96	59.00	0.32
	2017	1.18	0.72	0.34	54.40	0.16	9.70	45.49	0.34
		37%	4%	−6%	5%	−43%	8%	−23%	6%

Note: States are listed in ascending order of its percentage of population living under poverty line in 2011–12.

**Table A5:** Proportion of households with purchase records of the corresponding food group

	2013	2014	2015	2016	2017	Quarterly average from 2013 to 2017
Sweet snacks	0.95	0.95	0.96	0.96	0.97	0.89
Salty snacks	0.92	0.92	0.92	0.92	0.92	0.80
Soft drinks	0.72	0.70	0.69	0.70	0.69	0.46
Milk	0.99	0.99	0.99	0.99	0.99	0.96
Edible oils	0.91	0.92	0.92	0.92	0.92	0.90
Dairy products	0.74	0.72	0.72	0.73	0.73	0.53
Processed wheat	0.96	0.96	0.96	0.95	0.96	0.90
Other processed foods	0.90	0.91	0.87	0.87	0.90	0.73

**Table A6:** Difference in per capita annual purchase in urban India using NSSO data and ‘Kantar – Worldpanel Division, India’ data

	NSSO* (2011–12)	'Kantar – Worldpanel Division, India' (2013)	Estimation difference ('Kantar – Worldpanel Division, India'/NSSO)
Cold Beverages - bottled/canned (ml)	984	898ˆ	0.91
Edible oils (g)	10236	10903	1.07
Milk in liquid form (ml)	65064	65578	1.01
Butter (g)	48	85	1.77
Ghee (g)	600	669	1.11

*Calculated based 30-day per capita consumption reported by NSSO (NSSO report: http://mospi.nic.in/sites/default/files/publication_reports/Report_no558_rou68_30june14.pdf) ˆEstimates based on the annual purchase of carbonated drinks.

**Table A7:** Estimates of total urban population in India based on Indian Census and World Bank (WB) database, 2011–2017

	Estimates from Indian Census*	Estimates from WB	Difference (Indian Census/WB)
2011	377,106,125	390,085,540	0.97
2012	387,663,731	399,558,252	0.97
2013	398,516,913	409,178,263	0.97
2014	409,673,943	419,003,394	0.98
2015	421,143,331	429,068,623	0.98
2016	432,933,820	439,386,539	0.99
2017	445,054,399	449,964,523	0.99

Note: With the exception of 2011, the population figures were estimated using the annual population growth rate calculated from Indian Census (2001), 2011. The population estimates from WB are available at https://data.worldbank.org/indicator/SP.URB.TOTL?locations=IN.

## References

[bib1] Baker P., Friel S. (2014). Processed foods and the nutrition transition: evidence from Asia. Obes. Rev..

[bib2] Bandy L., Adhikari V., Jebb S., Rayner M., de Souza R.J. (2019). The use of commercial food purchase data for public health nutrition research: a systematic review. PLOS ONE.

[bib3] Borkotoky K., Unisa S. (2018). Inequality in food expenditure in India and the contributing factors. J. Quant. Econ..

[bib4] Caillavet F., Darmon N., Létoile F., Nichèle V. (2018). “Is nutritional quality of food-at-home purchases improving? 1969–2010: 40 years of household consumption surveys in France. Eur. J. Clin. Nutr..

[bib5] Cavatorta E., Shankar B., Flores-Martinez A. (2015). Explaining cross-state disparities in child nutrition in rural India. World Dev..

[bib49] Census of India (2011), Government of India, New Delhi.

[bib6] Cornelsen L., Mazzocchi M., Smith R.D. (2019). Fat tax or thin subsidy? How price increases and decreases affect the energy and nutrient content of food and beverage purchases in Great Britain. Soc. Sci. Med..

[bib7] Daniel C.R., Prabhakaran D., Kapur K., Graubard B.I., Devasenapathy N., Ramakrishnan L., George P.S., Shetty H., Ferrucci L.M., Yurgalevitch S., Chatterjee N., Reddy K., Rastogi T., Gupta P.C., Mathew A., Sinha R. (2011). A cross-sectional investigation of regional patterns of diet and cardio-metabolic risk in India. Nutr. J..

[bib8] Deaton A., Drèze J. (2009). Food and nutrition in India: facts and interpretations. Econ. Pol. Wkly..

[bib9] Development Initiatives (2018). 2018 Global Nutrition Report: shining a light to spur action on nutrition. https://globalnutritionreport.org/reports/global-nutrition-report-2018/.

[bib10] Drewnowski A., Popkin B.M. (1997). The nutrition transition: new trends in the global diet. Nutr. Rev..

[bib11] Euromonitor (2019). Passport Global Market Information Database.

[bib12] Ganguli D., Das N., Saha I., Biswas P., Datta S., Mukhopadhyay B., Chaudhuri D., Ghosh S., Dey S. (2011). Major dietary patterns and their associations with cardiovascular risk factors among women in West Bengal, India. Br. J. Nutr..

[bib13] Government of India (2014). Agricultural statistics at a glance. https://eands.dacnet.nic.in/PDF/Agricultural-Statistics-At-Glance2014.pdf.

[bib14] Government of India (2017). Notification No.1/2017-Compensation cess (rate. https://cbec-gst.gov.in/pdf/compensation-tax/notfctn-1-compensation-cess-english.pdf.

[bib15] Government of India (2017). Rate OF GST ON goods. http://gstcouncil.gov.in/sites/default/files/NOTIFICATION%20PDF/goods-rates-booklet-03July2017.pdf.

[bib16] von Grebmer K., Bernstein J., Hammond L., Patterson F., Klaus L., Fahlbusch J., Towey O., Foley C., Gitter S., Eckstrom K., Fritschel H. (2018). 2018 Global Hunger Index: Forced Migration und Hunger.

[bib17] Green R., Milner J., Joy E.J.M., Agrawal S., Dangour A.D. (2016). Dietary patterns in India: a systematic review. Br. J. Nutr..

[bib18] Gulati S., Misra A. (2017). Abdominal obesity and type 2 diabetes in Asian Indians: dietary strategies including edible oils, cooking practices and sugar intake. Eur. J. Clin. Nutr..

[bib19] Gulati S., Misra A. (2014). Sugar intake, obesity, and diabetes in India. Nutrients.

[bib20] Hawkes C., Harris J., Gillespie S. (2017). Changing diets: urbanization and the nutrition transition. 2017 Global Food Policy Report.

[bib21] IFT (2016). Global savory snacks market to reach $138+ billion by 2020 - IFT.org. http://www.ift.org/food-technology/daily-news/2016/september/07/global-savory-snacks-market-to-reach-$138-billion-by-2020.aspx.

[bib22] Kearney J. (2010). Food consumption trends and drivers. Philos. Trans. R. Soc. Lond. Ser. B Biol. Sci..

[bib23] Luhar S., Mallinson P.A.C., Clarke L., Kinra S. (2018). Trends in the socioeconomic patterning of overweight/obesity in India: a repeated cross-sectional study using nationally representative data. BMJ open.

[bib24] Meenakshi J.V. (2016). Trends and patterns in the triple burden of malnutrition in India. Agric. Econ..

[bib25] Misra A., Singhal N., Sivakumar B., Bhagat N., Jaiswal A., Khurana L. (2011). Nutrition transition in India: secular trends in dietary intake and their relationship to diet-related non-communicable diseases. J. Diabetes.

[bib26] Monteiro C.A., Moubarac J.-C., Cannon G., Ng S.W., Popkin B. (2013). Ultra-processed products are becoming dominant in the global food system. Obes. Rev..

[bib27] Moodie R., Stuckler D., Monteiro C., Sheron N., Neal B., Thamarangsi T., Lincoln P., Casswell S. (2013). Profits and pandemics: prevention of harmful effects of tobacco, alcohol, and ultra-processed food and drink industries. The Lancet.

[bib28] NITI Aayog (2018). Healthy States, Progressive India: Report on the Ranks of States and Union Territories | NITI Aayog, (National Institution for Transforming India), Government of India. https://niti.gov.in/content/healthy-states-progressive-india-report-ranks-states-and-union-territories.

[bib29] NSSO (2014). “Nutritional intake in India, 2011‐12, NSS 68th round. http://mospi.nic.in/sites/default/files/publication_reports/nss_report_560_19dec14.pdf.

[bib50] Pingali Prabhu, Mittra Bhaskar, Rahman Andaleeb (2017). The bumpy road from food to nutrition security–Slow evolution of India's food policy. Global food security.

[bib30] Popkin B., Ng S.W. (2007). The nutrition transition in high- and low-income countries: what are the policy lessons?. Agric. Econ..

[bib31] Popkin B.M. (2006). Global nutrition dynamics: the world is shifting rapidly toward a diet linked with noncommunicable diseases. Am. J. Clin. Nutr..

[bib32] Popkin B.M. (2014). Nutrition, agriculture and the global food system in low and middle income countries. Food Policy.

[bib33] Popkin B.M. (2017). Relationship between shifts in food system dynamics and acceleration of the global nutrition transition. Nutr. Rev..

[bib34] Popkin B.M., Adair L.S., Ng S.W. (2012). Global nutrition transition and the pandemic of obesity in developing countries. Nutr. Rev..

[bib35] Popkin B.M., Horton S., Kim S., Mahal A., Shuigao J. (2001). Trends in diet, nutritional status, and diet-related noncommunicable diseases in China and India: the economic costs of the nutrition transition. Nutr. Rev..

[bib36] Prabhakaran D., Jeemon P., Sharma M., Roth G.A., Johnson C., Harikrishnan S., Gupta R., Pandian J.D., Naik N., Roy A., Dhaliwal R.S., Xavier D., Kumar R.K., Tandon N., Mathur P., Shukla D.K., Mehrotra R., Venugopal K., Kumar G.A., Varghese C.M., Furtado M., Muraleedharan P., Abdulkader R.S., Alam T., Anjana R.M., Arora M., Bhansali A., Bhardwaj D., Bhatia E., Chakma J.K., Chaturvedi P., Dutta E., Glenn S., Gupta P.C., Johnson S.C., Kaur T., Kinra S., Krishnan A., Kutz M., Mathur M.R., Mohan V., Mukhopadhyay S., Nguyen M., Odell C.M., Oommen A.M., Pati S., Pletcher M., Prasad K., V Rao P., Shekhar C., Sinha D.N., Sylaja P.N., Thakur J.S., Thankappan K.R., Thomas N., Yadgir S., Yajnik C.S., Zachariah G., Zipkin B., Lim S.S., Naghavi M., Dandona R., Vos T., Murray C.J.L., Reddy K.S., Swaminathan S., Dandona L. (2018). “The changing patterns of cardiovascular diseases and their risk factors in the states of India: the Global Burden of Disease Study 1990–2016. The Lancet Global Health.

[bib37] Satija A., Hu F.B., Bowen L., V Bharathi A., Vaz M., Prabhakaran D., Reddy K.S., Ben-Shlomo Y., Davey Smith G., Kinra S., Ebrahim S. (2015). Dietary patterns in India and their association with obesity and central obesity. Publ. Health Nutr..

[bib38] Sen C.T. (2004). Food Culture in India.

[bib39] Shetty P. (2013). Nutrition transition and its health outcomes. Indian J. Pediatr..

[bib40] Shetty P.S. (2002). Nutrition transition in India. Publ. Health Nutr..

[bib41] The Economic Times (2016). “Kerala: in a first, Kerala imposes 14.5% ‘fat tax’ on junk food - the Economic Times. https://economictimes.indiatimes.com/news/politics-and-nation/in-a-first-kerala-imposes-14-5-fat-tax-on-junk-food/articleshow/53113799.cms.

[bib42] The New Indian Express (2019). “Kerala's action plan against trans fat within this week. https://www.fssai.gov.in/dam/jcr:65396482-4792-4b12-bd80-037292e9627d/FSSAI_News_Kerala_Express_11_02_2019.pdf.

[bib43] The Times of India (2017). Maggi market share: Maggi noodles has cornered 60% market share: nestle India CMD - Times of India. https://timesofindia.indiatimes.com/business/india-business/maggi-noodles-has-cornered-60-market-share-nestle-india-cmd/articleshow/56838663.cms.

[bib44] Thow A.M., Hawkes C. (2009). The implications of trade liberalization for diet and health: a case study from Central America. Glob. Health.

[bib45] Thow A.M., Kadiyala S., Khandelwal S., Menon P., Downs S., Reddy K.S. (2016). Toward food policy for the dual burden of malnutrition. Food Nutr. Bull..

[bib46] World Bank (2019). World Bank development indicators. https://data.worldbank.org/country/india.

[bib47] World Health Organization (2003). Diet, nutrition, and the prevention of chronic diseases: report of a joint WHO/FAO expert consultation. https://books.google.co.uk/books?hl=en&lr=&id=S6YsDwAAQBAJ&oi=fnd&pg=PA4&dq=Diet,+nutrition+and+the+prevention+of+chronic+diseases+-&ots=t8XSmoQFFh&sig=9JUU92rnsKFh7kR952SFWlINNjY.

[bib48] World Health Organization (2018). Obesity and overweight. https://www.who.int/en/news-room/fact-sheets/detail/obesity-and-overweight.

